# Combination Effects of Peramivir and Favipiravir against Oseltamivir-Resistant 2009 Pandemic Influenza A(H1N1) Infection in Mice

**DOI:** 10.1371/journal.pone.0101325

**Published:** 2014-07-03

**Authors:** Sehee Park, Jin Il Kim, Ilseob Lee, Sangmoo Lee, Min-Woong Hwang, Joon-Yong Bae, Jun Heo, Donghwan Kim, Seok-Il Jang, Hyejin Kim, Hee Jin Cheong, Jin-Won Song, Ki-Joon Song, Luck Ju Baek, Man-Seong Park

**Affiliations:** 1 Department of Microbiology, College of Medicine, and the Institute for Viral Diseases, Korea University, Seoul, Republic of Korea; 2 Department of Microbiology, College of Medicine, Hallym University, Chuncheon, Gangwon-do, Republic of Korea; 3 Division of Infectious Diseases, Korea University Guro Hospital, College of Medicine, Korea University, Seoul, Republic of Korea; The University of Chicago, United States of America

## Abstract

Antiviral drugs are being used for therapeutic purposes against influenza illness in humans. However, antiviral-resistant variants often nullify the effectiveness of antivirals. Combined medications, as seen in the treatment of cancers and other infectious diseases, have been suggested as an option for the control of antiviral-resistant influenza viruses. Here, we evaluated the therapeutic value of combination therapy against oseltamivir-resistant 2009 pandemic influenza H1N1 virus infection in DBA/2 mice. Mice were treated for five days with favipiravir and peramivir starting 4 hours after lethal challenge. Compared with either monotherapy, combination therapy saved more mice from viral lethality and resulted in increased antiviral efficacy in the lungs of infected mice. Furthermore, the synergism between the two antivirals, which was consistent with the survival outcomes of combination therapy, indicated that favipiravir could serve as a critical agent of combination therapy for the control of oseltamivir-resistant strains. Our results provide new insight into the feasibility of favipiravir in combination therapy against oseltamivir-resistant influenza virus infection.

## Introduction

Influenza viruses cause severe respiratory diseases. Vaccines and antiviral drugs can mitigate viral manifestations in humans. Compared with egg-based vaccines, which are known to be only effective against the target virus and are often faced with challenges, such as antigenic mismatch, substandard efficacy, and scaling-up manufacturing [1], antiviral drugs demonstrate broad-spectrum effectiveness against various subtypes of influenza viruses. Two classes of antiviral drugs are currently licensed in humans against influenza infections. One class comprises the amantadine analogs, amantadine hydrochloride and rimantadine, which inhibit virus replication by inhibiting a proton (H^+^) transport channel formed by the M2 protein and facilitating the failure of the release of the ribonucleoprotein (RNP) complex from the attached M1 protein into the cytoplasm [2], [3]. The other class targets the viral neuraminidase (NA) protein. Zanamivir and oseltamivir are well-known NA inhibitors (NAIs). By blocking NA enzymatic activity, these NAIs prevent a virus from invading through the respiratory mucins or newly assembled progeny virions in infected cells from budding out to neighboring cells [4].

The emergence of antiviral-resistant strains, however, hinders the common use of antiviral drugs in the treatment of influenza patients [5]. By achieving one amino acid mutation or additional accompanied mutations in the targeted viral protein [6], [7], influenza viruses can gain resistance to antiviral drugs without compromising viral fitness [8], [9] or transmissibility [10]–[12]. Changes in the glycosylation pattern of surface proteins may also contribute to immune evasion of antiviral resistant strains [13], [14]. Another concern is the enhanced pathogenicity observed in some of the antiviral-resistant, highly pathogenic influenza A H5N1 viruses [15], [16]. To alleviate their prevalence and subsequent pandemic potential, solid intervention methods or new approaches with broad-spectrum efficacy should be given priority over any other approaches [17]–[19].

Combination therapy is considered one of the strategies for addressing the antiviral resistance of influenza viruses. As seen in the treatment of HIV patients [20], the use of two or more antiviral drugs effective against different viral mechanisms or components enables evasion of the risks of resistant strains under the additive or synergistic efficacy of chemical combinations [21], [22]. The reduction of clinical complications and other resistance-related disease burdens can be another benefit of using combination therapy [23].

Peramivir, a newly introduced NAI that has a cyclopentane structure, has displayed *in vitro* and *in vivo* efficacy against influenza viruses. Its efficacy in treating influenza is attributed to its sustained residual plasma level [24], [25]. Currently, intravenous peramivir monotherapy has been licensed in Japan and South Korea. Favipiravir, formerly known as T-705, is another investigational drug that negatively affects the synthesis of influenza virus RNA through inhibition of the RNA-dependent RNA polymerase complex [26]. Compared with ribavirin, which also inhibits influenza polymerase activity, favipiravir exhibits less toxicity to host cells themselves and delivers better anti-influenza activity [27], [28]. In addition, favipiravir displays outstanding therapeutic effects in infected mice [29]. In a recent *in vivo* study of combination chemotherapy, these two agents exhibited a synergism and saved mice from lethal challenge with the 2009 pandemic H1N1 virus [30].

In the present study, we investigated the combination efficacy of peramivir and favipiravir against oseltamivir-resistant 2009 pandemic H1N1 virus infection in a DBA/2 mouse model, which was recently validated for the influenza antiviral screening study [31]. To compare with monotherapy, the mice were treated with the various combination sets of chemotherapy. The viral replication rates in the lungs were then determined in relation to the survival rates of mice after lethal challenge.

## Materials and Methods

### Ethics statement

The mouse experiments were conducted in accordance with the recommendations in the Guide for the Care and Use of Laboratory Animals of the Animal, Plant, and Fisheries Quarantine and Inspection Agency of Korea. The experimental protocols were approved by the Institutional Animal Care and Use Committee of Hallym University (permit number: Hallym 2012–22, 93).

### Cells and viruses

Madin-Darby canine kidney (MDCK) cells were obtained from American Type Culture Collection (Manassas, VA) and maintained in media supplemented with 10% fetal bovine serum and antibiotics for cell-based assays. A 2009 pandemic H1N1 virus (A/Korea/01/2009, K/09; NCBI taxonomy ID: 644289) and 2009 pandemic H1N1 oseltamivir-resistant variant A/Korea/2785/2009 (K/2785; see Table S1 for NA gene sequence information) virus harboring a NA H275Y (N1 numbering) mutation were provided by the Korea Centers for Disease Control and Prevention (KCDC, Osong, Korea). After being purified by a plaque assay in MDCK cells, the virus stocks were prepared from propagation in 10-day-old fertile chicken eggs. Reverse transcriptase-PCR and subsequent sequence analysis confirmed the NA H275Y mutation in the K/2785 virus, which confers resistance to oseltamivir. The rK09/NA:Y275 virus (see Table S1 for NA gene sequence information), which was previously rescued by plasmid-based reverse genetics [32], was also used as an oseltamivir-resistant control.

### Chemical compounds

Peramivir hydrate (Green Cross Corporation, Yongin, Korea) was obtained from the Division of Infectious Diseases, Korea University Guro Hospital (Seoul, Korea). Favipiravir and oseltamivir carboxylate were purchased from Adooq Bioscience (Irvine, CA) and Toronto Research Chemicals Inc. (Toronto, Ontario, Canada), respectively.

### Determination of IC_50_ and EC_50_ values

To determine the IC_50_ values of NAIs against influenza viruses, a modified fluorescence assay was performed using MU-NANA substrate [2′-(4-methylumbelliferyl)-a-D-N-acetylneuraminic acid; Sigma-Aldrich, St. Louis, MO]. Original virus samples were serially two-fold diluted, and the final substrate concentration was 100 µM. After a one-hour reaction with each NAI, the virus samples were incubated with the substrate for one hour. The released fluorescence was determined by SpectraMax M2e (Molecular Devices, LLC; Sunnyvale, CA), with excitation and emission wavelengths of 365 and 460 nm, respectively. The EC_50_ values were determined by plaque-reduction rates in MDCK cells. The data were then analyzed using Prism 5.0d software (GraphPad Software, Inc.; La Jolla, CA) by 95% confidence intervals.

### Mouse experiments

For body weight changes and survival rates observations, six- to seven-week-old female DBA/2 mice (Japan SLC, Inc., Hamamatsu, Japan) were infected intranasally with 2 MLD_50_ titer of the K/2785 virus, which was equivalent to 10^1.83^ plaque forming units (PFU). Intramuscular peramivir and/or oral favipiravir were administered twice daily (half daily dose per treatment) for five days starting from 4 hours post-infection (hpi). To assess the therapeutic efficacy of peramivir and/or favipiravir, DBA/2 mice were treated with the same protocols as described above. PBS was used for the mock infection and the placebo treatment in mice. Viral replication in the mouse lungs was then determined at seven and nine days post-infection (dpi) by plaque assay in MDCK cells. To determine the *in vivo* toxicity of chemicals, the body weight changes of mice treated twice daily (half daily dose per treatment) with peramivir (100 mg/kg/day) and favipiravir (40 mg/kg/day) for 5 days were recorded for 14 days post-treatment. To minimize animal suffering, mice were anesthetized intramuscularly with the combination of zoletile (10 mg/kg) and xylazine (15 mg/kg) before each viral infection and observed daily for their clinical changes (body weight, shivering, and fur condition). Mice exhibiting more than 25% weight loss were considered experimental death and euthanized humanely (cervical dislocation under deep anesthesia).

### Statistical analysis

Differences in the total number of survivors were evaluated by Fisher’s exact test using Prism 5.0d. Survival graphs were generated by Kaplan-Meier method and statistically analyzed with the Mantel-Cox log-rank test followed by the Gehan-Breslow-Wilcoxon test. Combination synergism was analyzed with MacSynergy II software [33] with 95% confidence limits. Differences in the lung viral titers of infected mice were evaluated by one-way ANOVA test and confirmed by Tukey’s multiple comparison test.

## Results

### Antiviral efficacy of chemical monotherapy

First, we assessed the susceptibility of the K/2785 virus against antiviral agents using fluorescence and cell-based assays. As previously known with other 2009 pandemic H1N1 strains [34], the K/09 virus was sensitive to oseltamivir carboxylate and peramivir (5.07 nM IC_50_ for oseltamivir carboxylate and 1.38 nM IC_50_ for peramivir; Table 1). However, the recombinant K09/NA:Y275 virus, which was formerly used as an oseltamivir-resistant control harboring the H275Y mutation in the NA protein (Table S1) [32], circumvented the antiviral efficacy of both NAIs, resulting in more than 75-fold elevated IC_50_ values compared with those of the K/09 virus (423.10 nM for oseltamivir carboxylate and 104.40 nM for peramivir; Table 1). The K/2785 virus which retaining the NA H275Y mutation was also resistant to NAIs and needed more than 20-fold higher concentrations (238 nM for oseltamivir carboxylate and 29.76 nM for peramivir) of NAIs to be controlled compared with the K/09 virus (Table 1). Unlike the limited efficacy of NAIs, a polymerase inhibitor, favipiravir, displayed a wider effectiveness against tested viruses. Only 8.39–14.66 µM of favipiravir was sufficient to restrict viral replication regardless of the presence of the H275Y mutation in the viral NA protein (Table 1).

**Table 1:** Viral susceptibility to antiviral agents.

	IC_50_ (nM)^a^		EC_50_ (µM)^b^
	Oseltamivir carboxylate	Peramivir	Favipiravir
K09	5.07 (3.58–7.17)	1.38 (0.70–2.74)	8.39 (3.76–18.74)
rK09/NA:Y275	423.10 (331.20–540.40)	104.40 (61.43–177.50)	14.66 (6.54–32.86)
K/2785	238 (127.70–443.60)	29.76 (15.55–56.94)	10.82 (5.93–19.76)

a IC_50_ values were determined by fluorescent NI assay.

b EC_50_ values were determined by plaque-reduction assay.

**Figure 1:**
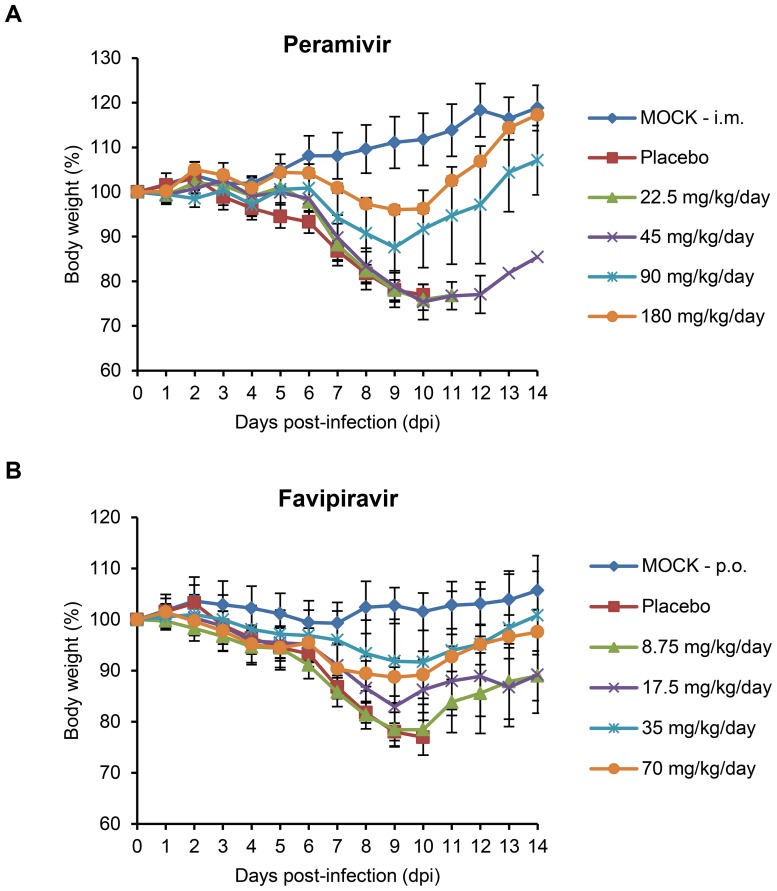
Effects of monotherapy with peramivir or favipiravir on mean body weight in mice infected with the oseltamivir-resistant K/2785 (H1N1) virus. Seven mice per each infection group were infected with 2 MLD_50_ titer of the K/2795 virus. Peramivir was administered by intramuscular (i.m.) injection (A), and favipiravir was administered orally (p.o.) (B). Chemicals (22.5, 45, 90, and 180 mg/kg/day for peramivir and 8.75, 17.5, 35, and 70 mg/kg/day for favipiravir) were maintained twice daily (half daily dose per treatment) for five days starting from 4 hours post-infection (hpi). Changes in the body weights are represented by the mean values ± standard deviations (SD). Mice in mock and placebo groups were infected or treated with PBS, respectively. The accompanying survival data are presented in Table 2.

We then investigated whether chemical monotherapy could protect mice from lethal influenza virus challenge. After infection with 2 MLD_50_ of the K/2785 virus, mice were treated with either peramivir (22.5, 45, 90, and 180 mg/kg/day) or favipiravir (8.75, 17.5, 35, and 70 mg/kg/day). By fatal challenge, mice were succumbed to death from 8 dpi (Figure 1 and Table 2). As the IC_50_ values indicated, peramivir presented unsatisfactory efficacy against oseltamivir-resistant virus infection. Most of the infected mice experienced severe weight loss (Figure 1A), and fatal outcomes were observed for all of the tested peramivir concentrations before 14 dpi (Table 2). With favipiravir, the infected mice also lost weight (Figure 1B), and only higher concentrations (35 or 70 mg/kg/day) saved more than 80% of the infected mice from death (Table 2). These results indicate that chemical monotherapy using either peramivir or favipiravir is insufficient to protect mice from lethal challenge of the oseltamivir-resistant K/2785 virus.

**Table 2:** Effects of monotherapy with peramivir or favipiravir on oseltamivir-resistant K/2785 virus infection in mice.

Favipiravir Peramivir		Survival/total	MDD^b^ ± SD
(mg/kg/day)^a^		(% survival)	(day)
Uninfected		7/7 (100.0%)	–
0	0	0/7 (0.0%)	10.0±0.8
0	22.5	0/7 (0.0%)	10.1±1.1
0	45	1/7 (14.3%)	10.2±0.4
0	90	5/7 (71.4%)*	10.5±2.1
0	180	5/7 (71.4%)*	11.0±1.4
8.75	0	2/7 (28.6%)	9.8±0.8
17.5	0	4/7 (57.1%)	10.0±0.0
35	0	6/7 (85.7%)**	11
70	0	7/7 (100.0%)***	–

a Intramuscular (peramivir) or oral (favipiravir) treatments were administered twice a day for five days starting from 4 hpi.

b MDD, mean day of death for mice that died prior to 14 dpi. Fisher’s exact test was applied to determine the statistical significance of differences in the survival rates (*, *P*<0.05, **, *P*<0.01, and ***, *P*<0.001 compared with the placebo mice group).

### Antiviral efficacy of combination therapy protocols

To mitigate the pathogenicity of the oseltamivir-resistant K/2785 virus in mice, various combination protocols were evaluated using peramivir and favipiravir (Table 3). The administration concentrations of each chemical were determined based on the results of the above monotherapy (25, 50, and 100 mg/kg/day for peramivir; 10, 20, and 40 mg/kg/day for favipiravir). PBS-infected (mock) or treated (placebo) mice were used as controls.

**Table 3:** Effects of combinations of peramivir and favipiravir on oseltamivir-resistant K/2785 virus infection in mice.

Favipiravir Peramivir		Survival/total	MDD^b^ ± SD	Lifespan increase^c^	Synergism^d^
(mg/kg/day)^a^		(% survival)	(day)	(day)	(%)
uninfected		6/6 (100.0%)	–		–
0	0	0/10 (00.0%)	9.4±1.0	–	0
	25	0/10 (00.0%)	9.8±1.5		0
	50	2/10 (20.0%)	10.1±1.2		0
	100	5/10 (50.0%)*	10.6±0.9		0
10	0	3/10 (30.0%)	9.4±0.5		0
	25	4/10 (40.0%)	10.5±0.5	0.7	10.0
	50	4/10 (40.0%)	10.7±1.2	0.6	−4.0
	100	5/10 (50.0%)*	11.2±1.0	0.6	−15.0
20	0	5/10 (50.0%)*	10.6±0.9		0
	25	7/10 (70.0%)**	9.7±0.6	0	20.0
	50	8/10 (80.0%)***	10.0±1.4	0	20.0
	100	8/10 (80.0%)***	11.0±1.4	0.4	5.0
40	0	8/10 (80.0%)***	10.7±0.6		0
	25	10/10 (100.0%)***	–	0	20.0
	50	10/10 (100.0%)***	–	0	16.0
	100	10/10 (100.0%)***	–	0	10.0

a See Table 2, footnote a.

b See Table 2, footnote b.

c Increase in lifespan of the infected mice was determined using the MDD results (the MDD of a combination therapy group compared with that of a longer living group between two respective monotherapy groups).

d Synergism was evaluated by MacSynergy II software. Fisher’s exact test was applied to determine the statistical significance of differences in the survival rates (*, *P*<0.05, **, *P*<0.01, and ***, *P*<0.001 compared with the placebo mice group).

**Figure 2:**
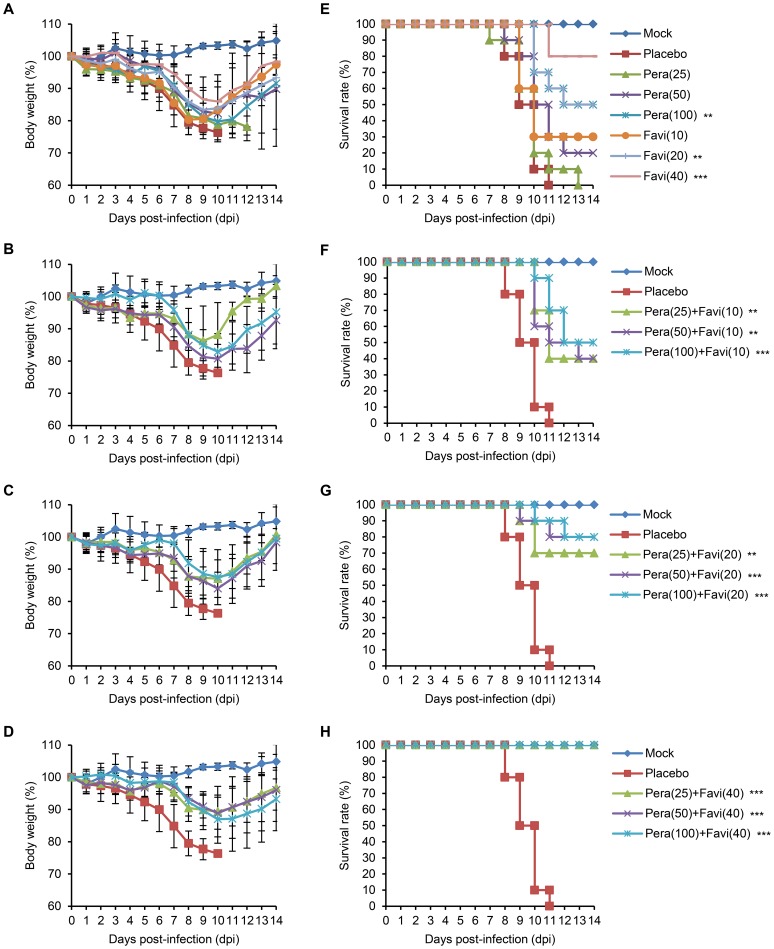
Effects of peramivir and favipiravir combination therapy for the treatment of the K/2785 virus-infected mice. 10 mice per group were infected and treated twice daily (half daily dose per treatment) with a monotherapy (A, E) of peramivir (25, 50, and 100 mg/kg/day) or favipiravir (10, 20, and 40 mg/kg/day) and a various set of combination therapy of peramivir and favipiravir (B, F for 10 mg/kg/day favipiravir groups; C, G for 20 mg/kg/day favipiravir groups; and D, H for 40 mg/kg/day favipiravir groups) for five days starting from 4 hpi. The body weight changes and survival rates were recorded for 14 days. The accompanying survival data are presented in Table 3. Survival graphs were generated by Kaplan-Meier method and statistically analyzed with the Mantel-Cox log-rank test followed by the Gehan-Breslow-Wilcoxon test (**, *P*<0.01, and ***, *P*<0.001 compared with placebo).

Compared with the PBS-infected controls, the infected mice in a placebo group exhibited severe clinical suffering, such as ruffled fur, shivering, and extreme weight loss (more than 25% of original body weight) (Figure 2A), and all succumbed to death between 8–12 dpi (Figure 2E). We again observed that peramivir alone was unable to produce sound therapeutic outcomes. Similar to the above monotherapy trials, 25, 50, and 100 mg/kg/day peramivir treatments cured less than half of the infected mice (0%, 20%, and 50% survival rates, respectively) (Figure 2A), and no increase in lifespan was observed (Table 3). However, combination therapy resulted in improved efficacy and gained life span increases in the infected mice. The same peramivir dosages, when coupled with 10 mg/kg/day favipiravir, reduced body weight loss in mice (Figure 2B) and resulted in respective 40%, 40%, and 50% survival rates (Figure 2F and Table 3). Interestingly, combination with increased concentrations of favipiravir produced even better therapeutic efficiencies. By producing less weight loss (Figure 2C and D), 20 or 40 mg/kg/day favipiravir saved more mice from lethal challenge, and the highest survival rates were 83.3% and 100%, respectively (Figure 2G and H and Table 3). The mean days until death were also lengthened with increasing dosage of favipiravir administered in combination therapy (Table 3), and no *in vivo* toxicity was observed in mice for the high dosage treatment of peramivir (100 mg/kg/day) and favipiravir (40 mg/kg/day) (data not shown). Considered together, these results indicate that the dearth in the mono-therapeutic efficacy of chemicals can be compensated for by using combination protocols, and an adequate combination formulation with favipiravir may enhance the survival rates of mice against oseltamivir-resistant K/2785 virus infection.

### Combination synergism between peramivir and favipiravir

**Figure 3:**
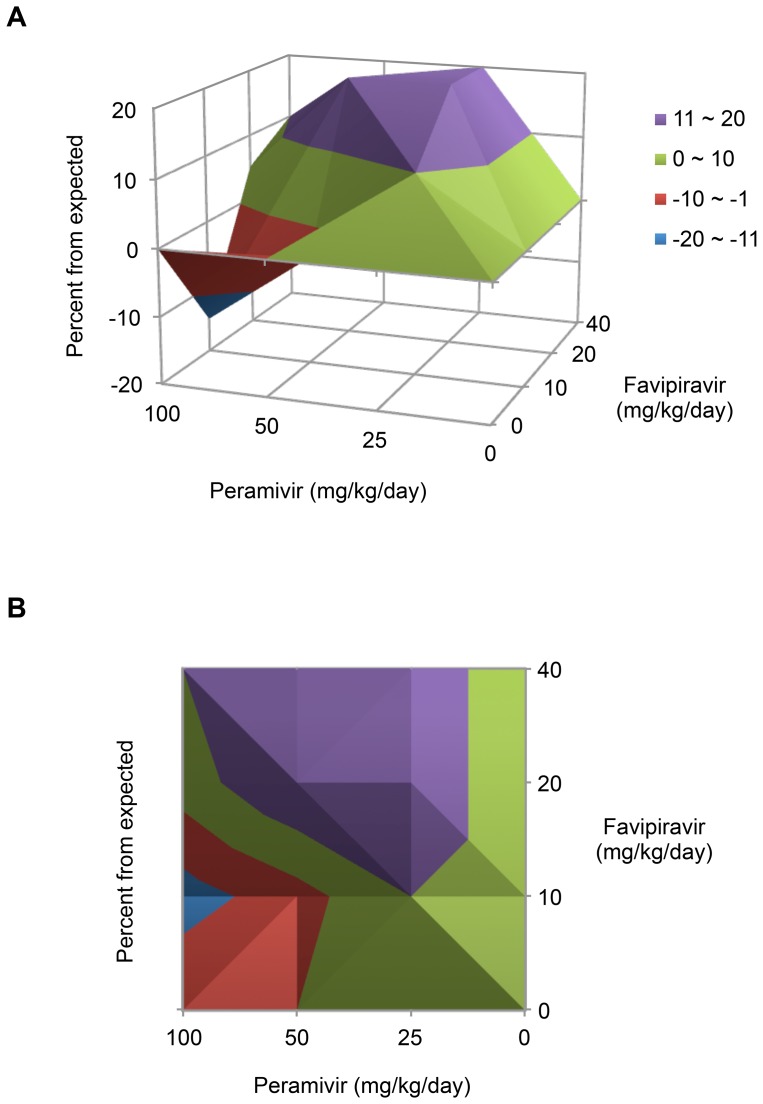
Combination synergism of peramivir and favipiravir. The interactions between peramivir and favipiravir based on the survival rates of the infected mice in Table 3 are represented by 3D (A) or 2D (B) plots generated after analysis with MacSynergy II software (Prichard and Shipman, 1990) [33].

Combination therapy protocols using peramivir and favipiravir resulted in different mechanisms of interaction. We then assessed the chemical synergism of these protocols using MacSynergy II software. In the three-dimensional (3D) representations, several treatment protocols were found to be synergistic when combined with 20 or 40 mg/kg/day favipiravir, whereas 10 mg/kg/day favipiravir was hardly effective even with 100 mg/kg/day peramivir (Figure 3A). In particular, 25 mg/kg/day peramivir established the greatest synergism (20.0 µm^2^ unit %) when combined with 20 (*P*<0.01) or 40 (*P*<0.001) mg/kg/day favipiravir (Figure 3A). 50 mg/kg/day peramivir also exhibited 20.0 µm^2^ unit % synergistic effect when combined with 20 mg/kg/day favipiravir (*P*<0.001) (Figure 3A). The net synergy was 82.0 µm^2^ unit %, and two combination sets (50 mg/kg/day peramivir and 10 mg/kg/day favipiravir, 100 mg/kg/day peramivir and 10 mg/kg/day favipiravir) displayed an antagonistic effect (−4.0 and −15.0 µm^2^ unit %, respectively) (Table 3). The 2D graph also represented the synergistic and antagonistic interactions between two chemicals (Figure 3B).

### Antiviral effects of combination therapy on the lungs of infected mice

**Figure 4:**
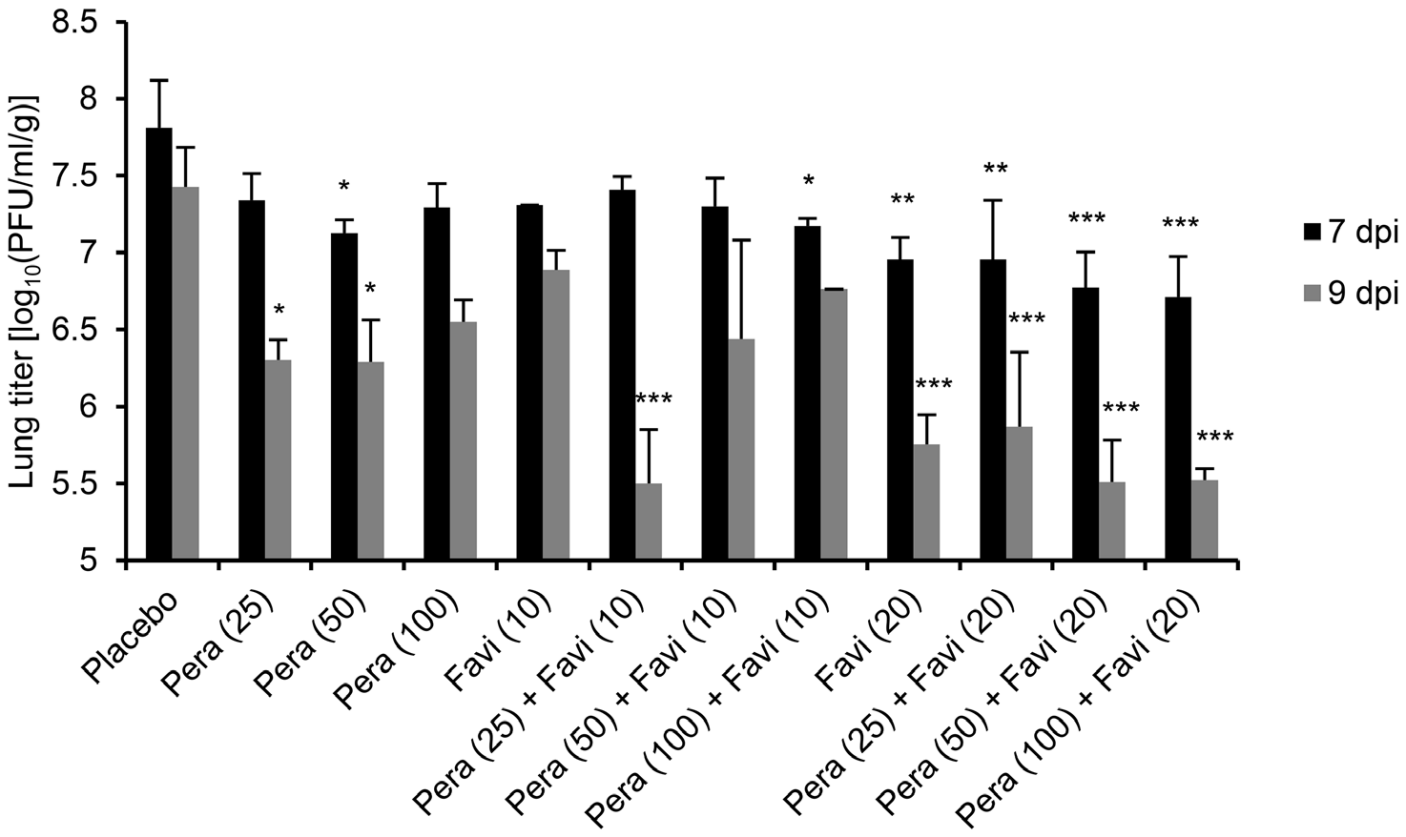
Therapeutic effects of combination therapy against viral replication in the lungs of infected mice. After infection and treatment, three mice were sacrificed at 7 and 9(± SD) were determined by three independent results. Exact viral titers were presented in Table 4. One-way ANOVA test was applied to analyze the statistical significance of differences in viral replication based on the used combination therapy, and Tukey’s multiple comparison test was performed (*, *P*<0.05, **, *P*<0.01, and ***, *P*<0.001 compared with placebo).

Severe pathogenesis in the lower respiratory tract is one of the most important etiologic features of influenza virus infection in humans. Therefore, we assessed whether combination therapy protocols using peramivir and favipiravir could counteract the K/2785 virus infiltration in the lungs of mice. In the lungs of the PBS-treated mice of a placebo group, the K/2785 virus was able to replicate up to 10^7.81±0.31^ and 10^7.42±0.26 ^PFU/ml per lung weight (g) by 7 and 9 dpi, respectively (Figure 4 and Table 4). These replication properties resulted in high pathogenesis of the K/2785 in mice. When treated, viral replication was alleviated according to the administered concentrations of the drugs. Generally, viral titer was reduced more by 9 dpi than by 7 dpi, and combination therapy was more efficacious than either peramivir or favipiravir monotherapy at protecting mice from lung viral invasion (Figure 4). In addition, the concentration of favipiravir but not that of peramivir appeared to be more critical in the drug combination because increases in peramivir concentrations did not reduce the severity of viral infiltration in the lungs, whereas favipiravir displayed dose-dependent protective efficacy (Figure 4 and Table 4). In particular, 20 mg/kg/day favipiravir combined with any peramivir concentration resulted in greater than one log reduction rates of viral replication in the lungs of infected mice (Figure 4 and Table 4), consistent with the chemical synergism observed in the previously examined combination data (Figure 3 and Table 3). These results indicate that combination therapy using peramivir and favipiravir has therapeutic potential against oseltamivir-resistant virus pathogenesis in mice.

**Table 4:** Viral titers in the lungs of infected mice after treatment.

Favipiravir Peramivir		Viral titers in lungs [log_10_(PFU/ml/g) ± SD]	
(mg/kg/day)^a^		7 dpi	9 dpi
0	0	7.81±0.31	7.42±0.26
	25	7.34±0.17	6.30±0.13*
	50	7.13±0.09*	6.29±0.27*
	100	7.29±0.15	6.55±0.14
10	0	7.31±0.01	6.88±0.12
	25	7.41±0.08	5.50±0.35***
	50	7.30±0.18	6.44±0.64
	100	7.17±0.05*	6.76
20	0	6.95±0.14**	5.75±0.19***
	25	6.96±0.38**	5.87±0.48***
	50	6.77±0.23***	5.51±0.28***
	100	6.70±0.27***	5.52±0.07***

a See Table 2, footnote a. Differences in the lung viral titers of infected mice were statistically evaluated by one-way ANOVA test and confirmed by Tukey’s multiple comparison test (*, *P*<0.05, **, *P*<0.01, and ***, *P*<0.001 compared with the placebo mice group).

## Discussion

Antiviral-resistant influenza viruses pose a significant threat to public health. Despite the fact that concerning potential events have yet to occur in the recurrent influenza seasons, a pandemic scenario involving the widespread distribution of antiviral-resistant variants always demands a comprehensive surveillance system for influenza [35]. The experimental results of *in vivo* transmission studies or simulations by mathematical modeling also underline the importance of medical preparedness for antiviral resistance [36], [37]. In fact, the somewhat limited modes of action-based but wide-spectrum antivirals are already available for the prevention and treatment of influenza infections in humans and have been stockpiled for urgent use. However, new lines of antivirals are still being pursued because the sustained use of the same or similar mechanism of drugs may instigate a high risk of the emergence of more potent outlying variants. To this end, peramivir is an alternative to existing NAI entries [24]. With comparable *in vitro* and *in vivo* efficacy to zanamivir and oseltamivir carboxylate, new NAIs based on cyclopentane structures have clinical potential [38]–[40]. The low emergence rates of resistant strains is another benefit aiding the prophylactic and therapeutic uses of peramivir [41]. However, there is a fraction of resistant variants that remain unmanaged.

We evaluated combination therapy using peramivir and favipiravir as a means of antiviral-resistant remedies in DBA/2 mice. As previously known, the combination of multiple agents can bring a series of therapeutic benefits, such as additive or synergistic inhibition of viral replication, less toxicity with reduced chemical doses, and limited resistance rates compared with chemical monotherapy [42]. Peramivir, together with ribavirin [43], rimantadine [44], [45], oseltamivir [46], or favipiravir [30], has been suggested as an effective component for combination therapy against various H1 and H5 strains [47]. In this study, peramivir alone lacked therapeutic efficacy against K/2785 virus infection in mice (Figures 1A and 2). It was also insufficient for the inhibition of viral replication in the infected lungs of mice (Figure 4 and Table 4). However, favipiravir appeared to be a useful choice for combination therapy against the oseltamivir-resistant K/2785 virus. Favipiravir (6-fluoro-3-hydroxy-2-pyrazinecarboxamide), an investigational drug formerly known as T-705, is an orally administered agent effective against cross-types of influenza viruses by targeting the viral RNA polymerase complex [48], [49]. Unlike ribavirin, which also blocks viral replication as a nucleoside inhibitor but inevitably harasses the viability of host cells, favipiravir is known to be safe from cytotoxicity and retains a high selectivity index [48]. For the mouse therapeutic model in our study, increases in the favipiravir concentration resulted in an improvement of survival rates (Table 3). Viral replication in the lungs was also controlled in a favipiravir concentration-dependent manner (Figure 4 and Table 4). Despite the disappointing performance of peramivir monotherapy, favipiravir exhibited synergistic interaction in combination therapy (Figure 3) that led to the overall survival enhancement of infected mice (Table 3). Considered together, these results indicate that favipiravir may be one of the essential agents for combination chemotherapy against various subtypes of influenza virus infections.

We used a DBA/2 mouse model to evaluate combination therapy. As suggested for human H1N1 viruses [50], DBA/2 mice were also highly susceptible to the K/2785 virus (MLD_50_ = 10^1.83^ PFU), which could avoid development of a lethal challenge virus after tedious rounds of adaptation in mice and lead us to focus on the virulence of the oseltamivir-resistant K/2785 virus itself, not on the genetic mutations considered when using mouse-adapted strains. As soon as the DBA/2 mice were infected with the lethal virus, they started to lose weight. Death was inevitable, and all of the infected mice succumbed to death from 8 dpi without treatment (Figures 1 and 2). Mono- or dual-chemotherapy demonstrated different therapeutic effects. At 7 dpi, viral pathogenesis resulted in more than 10% body weight loss, and mono- or dual-chemotherapy was less effective in protecting the infected lungs. However, viral replication at 9 dpi was far more reduced with the same treatments. In addition, the favipiravir-driven therapeutic effects of combination therapy were seen at 9 dpi (Figure 4 and Table 4), consistent with the results of the survival rate and combination synergism experiments (Figure 3 and Table 3).

We applied combination therapy against oseltamivir-resistant 2009 post-pandemic H1N1 virus infection in DBA/2 mice. Synergistically, the peramivir and favipiravir combination worked on the infected mice and protected them from severe viral pathogenesis. Despite the lack of clarity over whether the responsible NA H275Y mutation in the K/2785 virus occurred before or after human infection, this combination therapy has therapeutic potential in humans against naturally occurring oseltamivir-resistant H1N1 viruses. Furthermore, favipiravir is suggested as a key component of combination therapy to aid in the treatment of seasonal and pandemic influenza.

## Supporting Information

Table S1 **Comparison of viral NA gene sequences.**
(PDF)
