# Supplementary material for: Combination Effects of Peramivir and Favipiravir against Oseltamivir-Resistant 2009 Pandemic Influenza A(H1N1) Infection in Mice
Source: PLoS One. 2014 Jul 3;9(7):e101325. doi: 10.1371/journal.pone.0101325 (PMC4081560; doi:10.1371/journal.pone.0101325)
Supplement: Table S1 — Comparison of viral NA gene sequences. (PDF) [file pone.0101325.s001.pdf]

	10 20 30 40 50 60
K09	A T G A A T C C A A A C C A A A A G A T A A T A A C C A T T G G T T C G G T C T G T A T G A C A A T T G G A A T G G C T
rK09/NA:Y275	. . . .
K/2785	. . . .

	70 80 90 100 110 120
K09	A A C T T A A T A T T A C A A A T T G G A A A C A T A A T C T C A A T A T G G A T T A G C C A C T C A A T T C A A C T T
rK09/NA:Y275	. . . .
K/2785	. . . .

	130 140 150 160 170 180
K09	G G G A A T C A A A A T C A G A T T G A A A C A T G C A A T C A A A G C G T C A T T A C T T A T G A A A A C A A C A C T
rK09/NA:Y275	. . . .
K/2785	. . . .

	190 200 210 220 230 240
K09	T G G G T A A A T C A G A C A T A T G T T A A C A T C A G C A A C A C C A A C T T T G C T G C T G G A C A G T C A G T G
rK09/NA:Y275	. . . .
K/2785	. . . .

	250 260 270 280 290 300
K09	G T T T C C G T G A A A T T A G C G G G C A A T T C C T C T C T C T G C C C T G T T A G T G G A T G G G C T A T A T A C
rK09/NA:Y275	. . . .
K/2785	. . . . A . . . .

	310 320 330 340 350 360
K09	A G T A A A G A C A A C A G T A T A A G A A T C G G T T C C A A G G G G G A T G T G T T T G T C A T A A G G G A A C C A
rK09/NA:Y275	. . . .
K/2785	. . . .

	370 380 390 400 410 420
K09	T T C A T A T C A T G C T C C C C T T G G A A T G C A G A A C C T T C T T C T T G A C T C A A G G G G C C T T G C T A
rK09/NA:Y275	. . . .
K/2785	. . . .

	430 440 450 460 470 480
K09	A A T G A C A A A C A T T C C A A T G G A A C C A T T A A A G A C A G G A G C C C A T A T C G A A C C C T A A T G A G C
rK09/NA:Y275	. . . .
K/2785	. . . .

	490 500 510 520 530 540
K09	T G T C C T A T T G G T G A A G T T C C C T C T C C A T A C A A C T C A A G A T T T G A G T C A G T C G C T T G G T C A
rK09/NA:Y275	. . . .
K/2785	. . . .

```
             550                 560                 570                 580                 590                 600
K09          G C A A G T G C T T G T C A T G A T G G C A T C A A T T G G C T A A C A A T T G G A A T T T C T G G C C C A G A C A A T
rK09/NA:Y275 . . . . . . . . . . . . . . . . . . . . . . . . . . . . . . . . . . . . . . . . . . . . . . . . . . . . . . . . . . . .
K/2785       . . . . . . . . . . . . . . . . . . . . . . . . . . . . . . . . . . . . . . . . . . . . . . . . . . . . . . . . . . . .

             610                 620                 630                 640                 650                 660
K09          G G G G C A G T G G C T G T G T T A A A G T A C A A C G G A A T A A T A A C A G A C A C T A T C A A G A G T T G G A G A
rK09/NA:Y275 . . . . . . . . . . . . . . . . . . . . . . . . . . . . . . . . . . . . . . . . . . . . . . . . . . . . . . . . . . . .
K/2785       . . . . . . . . . . . . . . . . . . . . . . . . . . . . . C . . . . . . . . . . . . . . . . . . . . . . . . . . . . . .

             670                 680                 690                 700                 710                 720
K09          A A C A A T A T A T T G A G A A C A C A A G A G T C T G A A T G T G C A T G T G T A A A T G G T T C T T G C T T T A C T
rK09/NA:Y275 . . . . . . . . . . . . . . . . . . . . . . . . . . . . . . . . . . . . . . . . . . . . . . . . . . . . . . . . . . . .
K/2785       . . . . . . . . . . . . . . . . . . . . . . . . . . . . . . . . . . . . . . . . . . . . . . . . . . . . . . . . . . . .

             730                 740                 750                 760                 770                 780
K09          G T A A T G A C C G A T G G A C C A A G T A A T G G A C A G G C C T C A T A C A A G A T C T T C A G A A T A G A A A A G
rK09/NA:Y275 . . . . . . . . . . . . . . . . . . . . . . . . . . . . . . . . . . . . . . . . . . . . . . . . . . . . . . . . . . . .
K/2785       . . . . . . . . . . . . . . . . . . . . . G . . . . . . . . . . . . . . . . . . . . . . . . . . . . . . . . . . . . . .
                                                      N248D

             790                 800                 810                 820                 830                 840
K09          G G A A A G A T A G T C A A A T C A G T C G A A A T G A A T G C C C C T A A T T A T C A C T A T G A G G A A T G C T C C
rK09/NA:Y275 . . . . . . . . . . . . . . . . . . . . . . . . . . . . . . . . . . . . . . . . . . T . . . . . . . . . . . . . . . . .
K/2785       . . . . . . . . . . . . . . . . . . . . . . . . . . . . . . . . . . . . . . . . . . T . . . . . . . . . . . . . . . . .
                                                                                        H275Y

             850                 860                 870                 880                 890                 900
K09          T G T T A T C C T G A T T C T A G T G A A A T C A C A T G T G T G T G C A G G G A T A A C T G G C A T G G C T C G A A T
rK09/NA:Y275 . . . . . . . . . . . . . . . . . . . . . . . . . . . . . . . . . . . . . . . . . . . . . . . . . . . . . . . . . . . .
K/2785       . . . . . . . . . . . . . . . . . . . . . . . . . . . . . . . . . . . . . . . . . . . . . . . . . . . . . . . . . . . .

             910                 920                 930                 940                 950                 960
K09          C G A C C G T G G G T G T C T T T C A A C C A G A A T C T G G A A T A T C A G A T A G G A T A C A T A T G C A G T G G G
rK09/NA:Y275 . . . . . . . . . . . . . . . . . . . . . . . . . . . . . . . . . . . . . . . . . . . . . . . . . . . . . . . . . . . .
K/2785       . . . . . . . . . . . . . . . . . . . . . . . . . . . . . . . . . . . . . . . . . . . . . . . . . . . . . . . . . . . .

             970                 980                 990                 1000                1010                1020
K09          A T T T T C G G A G A C A A T C C A C G C C C T A A T G A T A A G A C A G G C A G T T G T G G T C C A G T A T C G T C T
rK09/NA:Y275 . . . . . . . . . . . . . . . . . . . . . . . . . . . . . . . . . . . . . . . . . . . . . . . . . . . . . . . . . . . .
K/2785       . . . . . . . . . . . . . . . . . . . . . . . . . . . . . . . . . . . . . . . . . . . . . . . . . . . . . . . . . . . .

             1030                1040                1050                1060                1070                1080
K09          A A T G G A G C A A A T G G A G T A A A A G G A T T T T C A T T C A A A T A C G G C A A T G G T G T T T G G A T A G G G
rK09/NA:Y275 . . . . . . . . . . . . . . . . . . . . . . . . . . . . . . . . . . . . . . . . . . . . . . . . . . . . . . . . . . . .
K/2785       . . . . . . . . . . . . . . . . . . . . . . . . . . . . . . . . . . . . . . . . . . . . . . . . . . . . . . . . . . . .
```

										1090										1100										1110										1120										1130										1140
K09	A	G	A	A	C	T	A	A	A	A	G	C	A	T	T	A	G	T	T	C	A	A	G	A	A	A	C	G	G	T	T	T	T	G	A	G	A	T	G	A	T	T	T	G	G	G	A	T	C	C	G	A	A	C	G	G	A	T	G	G
rK09/NA:Y275	.	.	.	.	.	.	.	.	.	.	.	.	.	.	.	.	.	.	.	.	.	.	.	.	.	.	.	.	.	.	.	.	.	.	.	.	.	.	.	.	.	.	.	.	.	.	.	.	.	.	.	.	.	.	.	.	.	.	.	.
K/2785	.	.	.	.	.	.	.	.	.	.	.	.	.	.	.	.	.	.	.	.	.	.	.	.	.	.	.	.	.	.	.	.	.	.	.	.	.	.	.	.	.	.	.	.	.	.	.	.	.	.	.	.	.	.	.	.	.	.	.	.

										1150										1160										1170										1180										1190										1200
K09	A	C	T	G	G	G	A	C	A	G	A	C	A	A	T	A	A	C	T	T	C	T	C	A	A	T	A	A	A	G	C	A	A	G	A	T	A	T	C	G	T	A	G	G	A	A	T	A	A	A	T	G	A	G	T	G	G	T	C	A
rK09/NA:Y275	.	.	.	.	.	.	.	.	.	.	.	.	.	.	.	.	.	.	.	.	.	.	.	.	.	.	.	.	.	.	.	.	.	.	.	.	.	.	.	.	.	.	.	.	.	.	.	.	.	.	.	.	.	.	.	.	.	.	.	.
K/2785	.	.	.	.	.	.	.	.	.	.	.	.	.	.	.	.	.	.	.	.	.	.	.	.	.	.	.	.	.	.	.	.	.	.	.	.	.	.	.	.	.	.	.	.	.	.	.	.	.	.	.	.	.	.	.	.	.	.	.	.

										1210										1220										1230										1240										1250										1260
K09	G	G	A	T	A	T	A	G	C	G	G	G	A	G	T	T	T	T	G	T	T	C	A	G	C	A	T	C	C	A	G	A	A	C	T	A	A	C	A	G	G	G	C	T	G	G	A	T	T	G	T	A	T	A	A	G	A	C	C	T
rK09/NA:Y275	.	.	.	.	.	.	.	.	.	.	.	.	.	.	.	.	.	.	.	.	.	.	.	.	.	.	.	.	.	.	.	.	.	.	.	.	.	.	.	.	.	.	.	.	.	.	.	.	.	.	.	.	.	.	.	.	.	.	.	.
K/2785	.	.	.	.	.	.	.	.	.	.	.	.	.	.	.	.	.	.	.	.	.	.	.	.	.	.	.	.	.	.	.	.	.	.	.	.	.	.	.	.	.	.	.	.	.	.	.	.	.	.	.	.	.	.	.	.	.	.	.	.

										1270										1280										1290										1300										1310										1320
K09	T	G	C	T	T	C	T	G	G	G	T	T	G	A	A	C	T	A	A	T	C	A	G	A	G	G	G	C	G	A	C	C	C	A	A	A	G	A	G	A	A	C	A	C	A	A	T	C	T	G	G	A	C	T	A	G	C	G	G	G
rK09/NA:Y275	.	.	.	.	.	.	.	.	.	.	.	.	.	.	.	.	.	.	.	.	.	.	.	.	.	.	.	.	.	.	.	.	.	.	.	.	.	.	.	.	.	.	.	.	.	.	.	.	.	.	.	.	.	.	.	.	.	.	.	.
K/2785	.	.	.	.	.	.	.	.	.	.	.	.	.	.	.	.	.	.	.	.	.	.	.	.	.	.	.	.	.	.	.	.	.	.	.	.	.	.	.	.	.	.	.	.	.	.	.	.	.	.	.	.	.	.	.	.	.	.	.	.

										1330										1340										1350										1360										1370										1380
K09	A	G	C	A	G	C	A	T	A	T	C	C	T	T	T	T	G	T	G	G	T	G	T	A	A	A	C	A	G	T	G	A	C	A	C	T	G	T	G	G	G	T	T	G	G	T	C	T	T	G	G	C	C	A	G	A	C	G	G	T
rK09/NA:Y275	.	.	.	.	.	.	.	.	.	.	.	.	.	.	.	.	.	.	.	.	.	.	.	.	.	.	.	.	.	.	.	.	.	.	.	.	.	.	.	.	.	.	.	.	.	.	.	.	.	.	.	.	.	.	.	.	.	.	.	.
K/2785	.	.	.	.	.	.	.	.	.	.	.	.	.	.	.	.	.	.	.	.	.	.	.	.	.	.	.	.	.	.	.	.	.	.	.	.	.	.	.	.	.	.	.	.	.	.	.	.	.	.	.	.	.	.	.	.	.	.	.	.

										1390										1400										1410
K09	G	C	T	G	A	G	T	T	G	C	C	A	T	T	T	A	C	C	A	T	T	G	A	C	A	A	G	T	A	A
rK09/NA:Y275	.	.	.	.	.	.	.	.	.	.	.	.	.	.	.	.	.	.	.	.	.	.	.	.	.	.	.	.	.	.
K/2785	.	.	.	.	.	.	.	.	.	.	.	.	.	.	.	.	.	.	G	.	.	.	.	.	.	.	.	.	.	.
																				I467V										
